# Occurrence of mastitis pathogens in cow’s milk samples from the Hanover region, north-western Germany – an overview of routine laboratory data from 2005 to 2023

**DOI:** 10.1007/s11259-025-10773-1

**Published:** 2025-05-22

**Authors:** Theresa Büthe, Nadja Jessberger, Bettina Schneider, Lothar Kreienbrock, Madeleine Plötz

**Affiliations:** 1 Institute for Food Quality and Food Safety, University of Veterinary Medicine Hannover, Bischofsholer Damm 15, 30173 Hanover, Germany; 2 Institute of Biometry, Epidemiology and Information Processing, University of Veterinary Medicine Hannover, Bünteweg 2, 30559 Hanover, Germany

**Keywords:** Bacterial pathogens, Dairy industry, Longitudinal trend, Non-aureus staphylococci and mammaliicocci, *Streptococcus uberis*, *Escherichia coli*

## Abstract

Mastitis remains the most frequent disease in dairy cows, affecting both animal welfare and the profitability of dairy farms. In the present investigation, we retrospectively analysed 102,179 cow’s milk samples, which were object of routine diagnostics at the Department of Milk Hygiene, Institute for Food Quality and Food Safety, University of Veterinary Medicine Hannover, from 2005 to 2023, regarding the occurrence of major mastitis pathogens. On average, approx. 48% of all tested samples were bacteriologically positive. Differences in the occurrence of positive findings were described regarding the season, the submitter, the reason for submission, as well as the udder quarters. Generally, the 13 most common groups of pathogens accounted for 94.0% of all positive samples. Non-aureus staphylococci and mammaliicocci (23.6%) were detected most frequently, followed by *Streptococcus uberis* (14.8%), *Escherichia coli* (13.2%), *Corynebacterium* spp. (9.5%), *Staphylococcus aureus* (6.7%), yeast (6.1%), and *Enterococcus* spp. (5.6%). While a slight decline was recorded for most of these pathogen groups, *S. uberis* and *E. coli* showed a significant upward trend throughout the investigation period. Moreover, a constantly high percentage of samples contaminated with more than three different colony forms was found. Monitoring long-term development of the mastitis pathogen spectrum as well as its antibiotic resistance patterns is of utmost importance to enable adaptation of hygiene and treatment strategies. This includes appropriate training of the treating veterinarians with regard to sterile milk sampling.

**Supplementary Information:**

The online version contains supplementary material available at 10.1007/s11259-025-10773-1.

## Introduction

Germany is the largest producer of cow’s milk inside the European Union, followed by France and The Netherlands (Destatis 2024). Nearly half of the country’s production comes from the Federal states of Bavaria (approx. 25%) and Lower Saxony (approx. 22%). In the latter, 7,569 dairy farms and 783,835 dairy cows were domiciled in 2023 (LVN 2024).

Mastitis is the most common disease in dairy cows, which has a strong impact not only on animal wellbeing, but also on the profitability of dairy farms (Ruegg 2017). Despite no visible symptoms, the most prevalent, subclinical form has a massive effect on milk yield, milk and product quality and general herd productivity. It can also affect conception and fertility (Ruegg 2017; Ashraf and Imran 2020). Thus, frequent monitoring resulting in fast and early detection of mastitis and identification of the pathogen are of utmost importance.

Although the present investigation mainly focuses on key pathogens, almost 200 different microorganisms are described, which can cause bovine mastitis. These are primarily bacteria, but also viruses, fungi and yeast (Ashraf and Imran 2020). Classically, it is distinguished between contagious (e.g. *Staphylococcus aureus* and *Streptococcus agalactiae*) and environmental (e.g. *Streptococcus uberis*, *Escherichia coli*, the majority of non-aureus staphylococci and mammaliicocci (NASM) and enterococci) mastitis pathogens (Ruegg 2017). *S. aureus* is estimated to account for 50% of the global mastitis cases (El-Sayed and Kamel 2021). However, this classic categorization staggers, as many pathogens behave both ways. For example, *Prototheca* are transmitted from cow to cow and from the environment to cow, some NASM species can be contagious as well, and *Streptococcus dysgalactiae* and *S. uberis* may also act like contagious pathogens (Esener et al. 2018; Wente and Krömker 2020).

The present investigation summarizes results of the cow’s milk samples submitted for pathogen identification to the Department of Milk Hygiene, Institute for Food Quality and Food Safety, University of Veterinary Medicine Hannover. Data from 2005 to 2023, i.e. over a period of 19 years were available. These were samples obtained from routine examinations and herd management, as well as samples received from practicing veterinarians and our clinical facilities after illness of the animals. This investigation aims to analyze the pathogen spectrum and assess its temporal changes over a 19-year period.

## Materials and methods

### Sample collection

From 2005 to 2023, a total of 102,179 samples were obtained by our diagnostics laboratory either via personal delivery (veterinary stock supervisions, Ambulatory Veterinary Service for Large Animals, Clinic for Small Cloven-hoofed Animals and Clinic for Cattle at University of Veterinary Medicine Hannover) or via postal dispatch (practicing veterinarians). Supplementary Fig. 1 gives an overview of the submitters, the origin and the geographical allocation of the samples.

### Differentiation of mastitis-causing pathogens

All laboratory analyses were performed following the DVG (Deutsche Veterinärmedizinische Gesellschaft e. V.) guidelines for isolation and identification of mastitis pathogens (DVG 2009), which basically describe the same microbiological and biochemical differentiation tests as the NMC (National Mastitis Council) guidelines. Routinely, samples were checked for macroscopic alterations and subsequently, 10 µl were streaked each on esculin blood (Thermo Fisher Scientific) and YGC agar (yeast-glucose-chloramphenicol, Thermo Fisher Scientific), and added to 7 ml nutrient broth I (10 g/l Liebig meat extract (Merck), 10 g/l peptone from meat (Merck), 3 g/l NaCl (Thermo Fisher Scientific), 2 g/l Na₂HPO₄ x 12 H₂O (Merck), 10 g/l D(+)-glucose 1-hydrate (AppliChem GmbH), pH 7.4) using a sterile inoculation loop. Sterility of the growth media was ensured by applying non-inoculated negative controls. Nutrient broth I was incubated for 24 h at 37 °C and checked for bacterial growth and gas formation. After that, a 10 µl sample was streaked on a second esculin blood agar plate (Thermo Fisher Scientific), which was incubated for another 24 h at 37 °C. Blood and YGC agar plates were incubated for 48 h at 37 °C, and colony sizes and morphologies were evaluated after 24 and 48 h. Depending on colony size, color and morphology, pure or mixed bacterial cultures were differentiated. If more than three different colony morphologies were distinguishable, the sample was generally classified as “contaminated”. Depending on the amount of relevant mastitis pathogens on the blood agar (1/3, 2/3 or complete overgrowth), their content in the original milk sample was designated high, medium or low. Colonies visually suspicious for Enterobacteriaceae and especially *E. coli* were additionally streaked on Chromocult^®^ agar (Merck, specific for coliforms and *E. coli*) and incubated for 24 h at 37 °C. Samples to be tested for clostridia (on specific request or if suspected) were incubated under anaerobic conditions.

Esculin hydrolysis and occurrence of hemolysis were determined directly on the blood agar plates. For differentiation of yeast and *Prototheca* grown on YGC plates, native preparations (colony material solved in 10 µl 0.9% NaCl solution) were examined under a Standard 25 ICS microscope (Carl Zeiss AG) in 1000-fold magnification. Bacterial samples were treated similarly and additionally subjected to Gram staining. Cytochrome oxidase was detected with the Bactident^®^ oxidase strips (Merck) according to the instructions of the manufacturer. The Oxoid™ O.B.I.S. PYR kit (Oxoid; Thermo Fisher Scientific) was used for rapid detection of PYRase activity in streptococci or *Citrobacter* spp. following the instructions of the supplier. For the identification of streptococcal groups A, B, C, D, F and G according to Lancefield, the Streptococcal Grouping Kit using Latex Agglutination (Oxoid; Thermo Fisher Scientific) was applied following the instructions of the manufacturer. Furthermore, a hippurate test kit was used for the detection of hippurate hydrolysis according to the instructions of the manufacturer (Liofilchem S.r.l.). For differentiation of staphylococci and the identification of the “clumping factor”, the Staphylase™ agglutination rapid test (Oxoid; Thermo Fisher Scientific) was used according to the instructions of the producer. For further confirmation, a coagulase test was applied for differentiation between coagulase-positive and -negative staphylococci. For this, 2–4 colonies were added to 200 µl rabbit blood plasma (BBL™ Coagulase Plasma, rabbit with EDTA; Thermo Fisher Scientific). After four and additionally after 24 h of incubation at 37 °C, the suspension was checked for coagulation. Species were further differentiated by using Api^®^ ID 32 E test strips (for identification of Enterobacteriaceae and other Gram-negative rods; bioMérieux Deutschland GmbH) or by MALDI-TOF-MS analysis (not routinely, but applied to approx. 0.5% of all samples, upon special request of the customers, for research purposes or for further NASM differentiation) using the microflex LT/SH mass spectrometer (Bruker Daltonik GmbH) according to the manufacturer’s instructions.

### Data analysis

The raw data was collected over the years and re-structured within Microsoft Excel^®^, version 2016. For further statistical analyses, data was transferred to SAS^®^, version 9.4, TS level 1M5 (SAS Institute Inc.). Data was checked for general completeness for basic variables under investigation, i.e. a complete case-analysis was performed. Samples with less than 200 findings (i.e. less than 10 samples per year) were not considered for further analyses. Only basic data from the samples was used; additional information from the sample environment or clinical and other veterinary information was not registered at the lab and not considered therefore. Within this information, general explorative data analyses were performed without any statistical hypotheses testing to describe general outcome and distributions. The amount of positive outcome per year was described as linear trend with means of simple linear regression analysis.

## Results

### Distribution of positive and negative samples

**Fig. 1:**
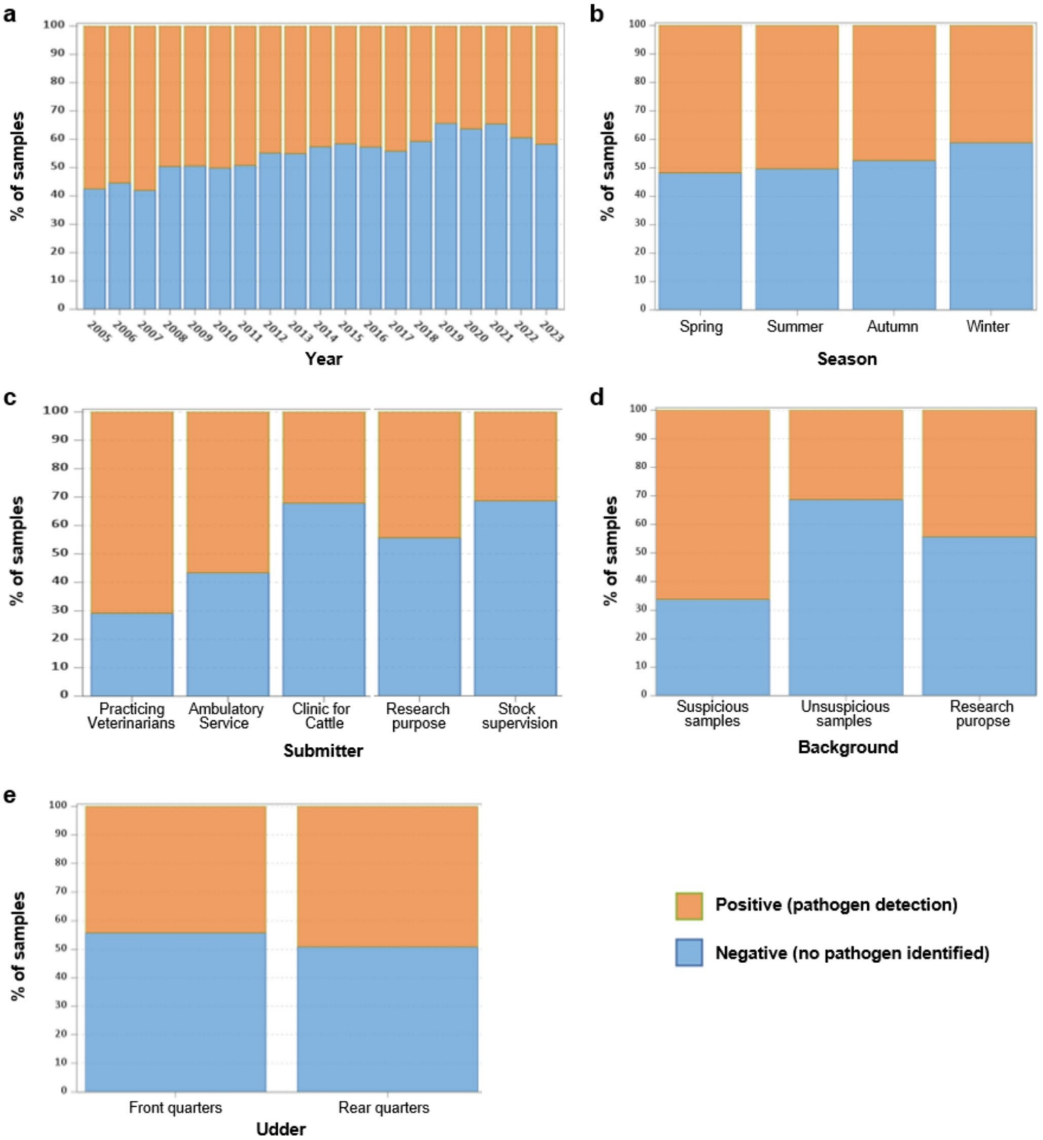
Distribution of positive (pathogen detection) and negative (no pathogen identified) samples, **a** by year, **b** by season (spring: March, April, May; summer: June, July, August; autumn: September, October, November; winter: December, January, February, **c** by groups of clients/submitters, **d** by background/reason for submission, **e** by udder quarters

From 102,179 submitted cow’s milk samples from 2005 to 2023, 48,818 (47.8%) were found positive (pathogen detection), and 53,361 (52.2%) negative (no pathogen identified). Figure 1A shows the development by year, indicating a decrease in the percentage of positive samples over the years. An analysis of the distribution across the different seasons reveals a slightly higher percentage of samples in which pathogens were detected in spring and summer (Fig. 1B).

It also became apparent that most positive results were found in samples, where a preliminary report or suspicion of disease in the animals already existed. This is also reflected in the statistics of the sample submitters (Fig. 1C). The highest numbers of positive samples were found among practising veterinarians, followed by the Ambulatory Veterinary Service for Large Animals. Both sent in samples primarily from a small number of animals per case, which suffered from mastitis. The lowest percentage of positive results was recorded in samples from stock supervision, i.e. routine checks of entire herds, as well as in samples from the Clinic for Cattle.

Furthermore, a comparison of the number of positive findings with the background/reason for sample submissions showed that pathogens were mainly detected in suspicious samples, e.g. reported illness of the animal, and least in unsuspicious samples, e.g. from herd management (Fig. 1D). Additionally, a slightly higher percentage of positive results was found in samples from the rear udder quarters compared to the front ones (Fig. 1E).

### Isolated microorganisms from dairy milk samples

Table 1 gives an overview of the identified pathogens accumulated from 2005 to 2023. Generally, the 25 most common groups of pathogens accounted for 98.3%, the 13 most common groups for 94.0% of all positive samples. Non-aureus staphylococci and mammaliicocci (NASM; 23.6% of total bacteriologically positive samples) were detected most frequently, followed by *S. uberis* (14.8%), *E. coli* (13.2%), *Corynebacterium* spp. (9.5%), *S. aureus* (6.7%), yeast (6.1%), and *Enterococcus* spp. (5.6%). Next most frequent were contaminated samples (more than three different colony forms; 5.0%), followed by *Bacillus* spp. (3.4%), *Streptococcus dysgalactiae* (2.7%), *Klebsiella pneumoniae *(1.5%), moulds (1.0%) and *Klebsiella ozaenae* (1.0%).

**Fig. 2:**
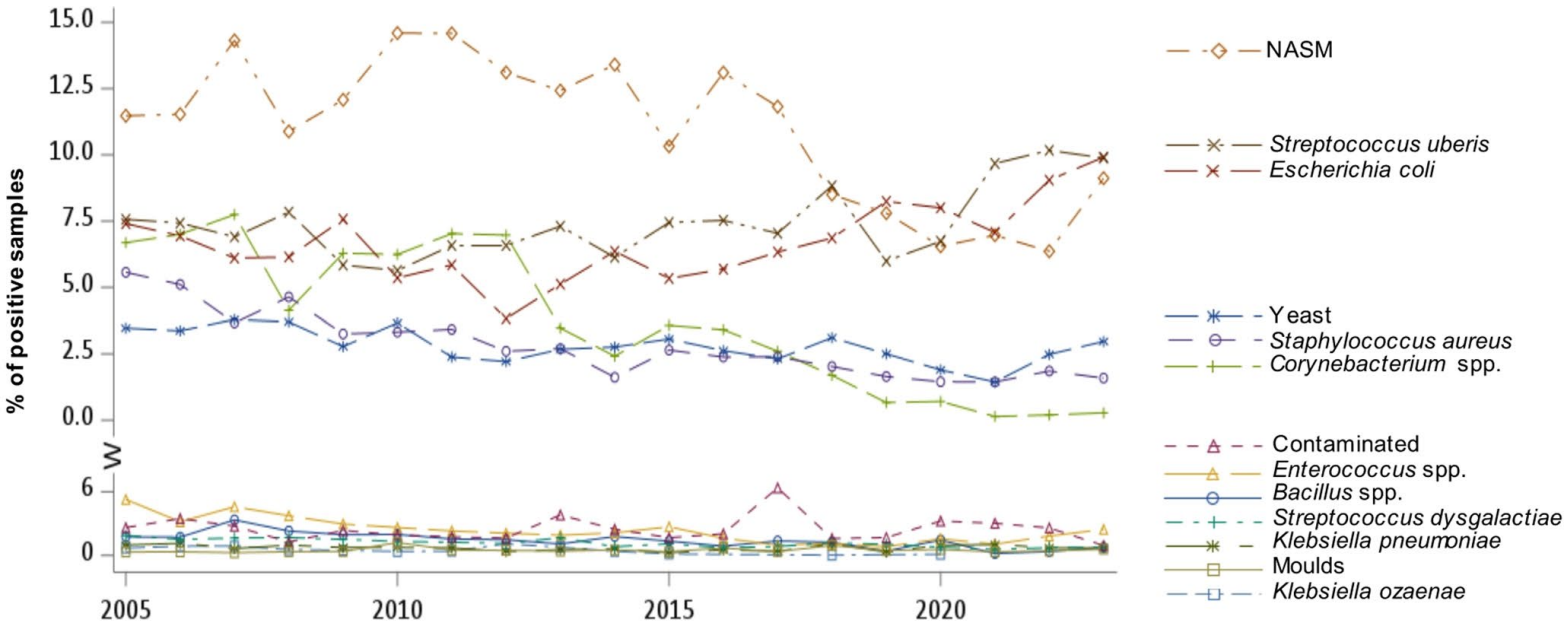
Proportion of the 13 most prevalent pathogen groups of all positive samples per year

In Fig. 2, the course of the frequencies of the 13 most common pathogen groups over the total investigation period is depicted. Slightly and statistically decreasing (linear) trends were observed over the years for NASM (*p* = 00066), *Corynebacterium* spp. (*p* < 0.0001), *S. aureus* (*p* < 0.0001), *Enterococcus* spp. (*p* < 0.0001), *Bacillus* spp. (*p* < 0.0001), *S. dysgalactiae* (*p* < 0.001), *K. ozaenae* (*p* = 0.01066) and yeast (0.0027), while *S. uberis* (*p* = 0.01904) and *E. coli* (*p* = 0.03448) were the only pathogens exhibiting a slight upward linear trend (Fig. 2 and Supplementary Table 1).

Moreover, a selection of approx. 300 NASM isolates randomly collected over the years was characterized at species level using MALDI-TOF-MS. Most frequently found was *S. chromogenes* (44.0%), followed by *M. sciuri* (20.6%), *S. xylosus* (10.7%), *S. haemolyticus* (7.2%), *S. epidermis* (3.8%), *S. borealis* (3.4%) and *S. simulans* (3.1%). *S. capitis*, *S. cohnii*, *S. equorum*, *S. fleurettii*, *S. gallinarum*, *S. lugdunensis*, *S. muscae*, *S. saprophyticus*, *S. haemolytica*, *S. vitulinus*, *S. warneri*, *S. hominis*, *S. hyicus* and *S. succinus* were only sporadically detected.

**Table 1:** Results of 102,179 cow’s milk samples during the investigation period from 2005 to 2023. The sample size, the percentage of total samples, as well as the percentage of positive samples is shown for the 25 most prevalent pathogen groups, sorted by frequency of occurrence. Others comprise all pathogens of which less than 100 isolates were found in total. These are, in descending order, *Klebsiella* spp., *Aerococcus viridans*, *Aeromonas* spp., *Pseudomonas aeruginosa*, *Enterobacter cloacae*, *Citrobacter koseri*, *Streptococcus* spp., *Prototheca zopfii*, *Streptococcus canis*, *Acinetobacter* spp., *Hafnia alvei*, *Pantoea agglomerans*, *Enterobacter* spp., *Citrobacter* sp., *Aeromonas* sp., *Serratia odorifera*, *Serratia* sp., *Streptomyces* spp., enterobacteriaceae, *Pasteurella multocida*, *Pasteurella* sp., *Streptococcus bovis*, *Clostridum* sp., non-differentiated streptococci of group D or E, *Micrococcus* spp., *Nocardia* sp., actinomycetes, *Lactococcus* spp., *Pasteurella haemolytica*, *Streptococcus equi zooepidemicus*, and *Streptococcus oralis*

Finding	Sample size	% of total samples	% of positive samples
Total	102,179	100.00	-
Negative	53,361	52.22	-
Positive	48,818	47.78	100.00
NASM	11,538	11.29	23.63
*Streptococcus uberis*	7,201	7.05	14.75
*Escherichia coli*	6,449	6.31	13.21
*Corynebacterium* spp.	4,650	4.55	9.53
*Staphylococcus aureus*	3,252	3.18	6.66
Yeast	2,975	2.91	6.09
*Enterococcus* spp.	2,750	2.69	5.63
Contaminated	2,434	2.38	4.99
*Bacillus* spp.	1651	1.62	3.38
*Streptococcus dysgalactiae*	1,284	1.26	2.63
*Klebsiella pneumoniae*	738	0.72	1.51
Moulds	478	0.47	0.98
*Klebsiella ozaenae*	469	0.46	0.96
*Trueperella pyogenes*	444	0.43	0.91
*Proteus* spp.	233	0.23	0.48
Coliforms	232	0.23	0.48
*Klebsiella oxytoca*	162	0.16	0.33
*Serratia marcescens*	153	0.15	0.31
*Klebsiella aerogenes*	149	0.15	0.31
*Citrobacter freundii*	147	0.14	0.30
Group D streptococci	137	0.13	0.28
*Flavobacterium* spp.	119	0.12	0.24
*Serratia liquefaciens*	114	0.11	0.23
*Pseudomonas* spp.	108	0.11	0.22
*Streptococcus agalactiae*	102	0.10	0.21
Others	849	0.83	1.74

## Discussion

The present investigation summarizes 19 years of routine diagnostics of cow’s milk samples at the University of Veterinary Medicine Hannover. The investigation therefore is a secondary data use of routine samples and with this appears not to be representative for the entire German dairy population. First, it has to be stated that most of the data collected is from the north-western part of Germany only. Here, Holstein-Friesian dairy cows are most popular, which restricts the results mainly to this breed and its related management system in general. Second, to a certain extent, the information collected is double selective, as most of the customers send in samples only after a disease is suspected. This on the one hand may overestimate prevalence. On the other hand, the laboratory itself offers selected tests only. It is therefore not possible to specify the exact prevalence of the detected pathogens within the target population of dairy production. However, the general organisation as an independent consulting laboratory with a sustainable and harmonised standard operating procedure guarantees valuable results in identifying single events and especially to view a longitudinal process, particularly in the Hanover region, where most of the samples (approx. 63%) originate.

From an epidemiological point of view, these restrictions can therefore reveal all three basic bias mechanisms, i.e. selection bias as described above, information bias due to the restrictions in the laboratory procedures and confounding bias due to missing information in many factors on farm, which are unknown in the database presented here. Therefore, no statistical hypotheses were tested with means of statistical test procedures and merely a data exploration is presented. Only for describing a longitudinal trend a linear regression by year is presented. However, the presented p-values again have to be interpreted as general description. All of the results shown here therefore have the character of generating hypotheses, which can be tested in more detail in further studies through specific investigations.

However, a first notable result was a higher percentage of positive samples in the spring and summer months, which might be due to enhancement of bacterial (pathogen) growth by higher temperatures, facilitated transmission of pathogens by vectors such as flies and other insects, higher exposure of the animals to the pathogens on the pasture, weakened immune system of the animals due to heat stress, or hygiene deficiencies due to increased contamination of the barns (Sharma et al. 2023; Stanek et al. 2024; Sommer et al. 2024). A more detailed hypothesis on this could not be tested due to missing details on the sample environment, management or other clinical information.

Furthermore, the rear quarters of the cows’ udders were slightly more often bacteriologically positive than the front quarters. Earlier studies also showed a higher prevalence of subclinical and clinical mastitis in the rear quarters (Adkinson et al. 1993; Berry and Meaney 2006). This might result from milking machine vacuum settings or milking routines, from the morphological structure of the udder, larger milk production in the rear quarters, their more frequent exposure to faecal contamination, injury or teat end damage, and depends on age and parity of the cow (Berry and Meaney 2006).

Generally, we most frequently found NASM (23.6%), *S. uberis* (14.8%), and *E. coli* (13.2%). In a study from 2015, more than 1 million test results from 11 milk laboratories in eight German federal states were evaluated. Most prevalent pathogens were *S. uberis* and other esculin-positive streptococci (30.5%), followed by NASM (28.5%), and *S. aureus* (12.4%) (DVG 2016). In a retrospective study, 85,979 samples were analysed, which were taken over a 10-year period from dairy cattle in Ontario, Canada. Major bacterial pathogens detected were *S. aureus* (15.6%), NASM (5.0%), *Corynebacterium* spp. (2.9%), and *E. coli* (2.0%). Furthermore, the prevalence of contagious pathogens such as *S. aureus* and *Corynebacterium* spp., as well as the prevalence of all major environmental mastitis pathogens except *T. pyogenes*, increased over time (Acharya et al. 2021). These findings coincide only partly with the results of our investigation, in which an upward trend was seen only for *S. uberis* and *E. coli*. Furthermore, the authors found proof for not only seasonal, but also geographical patterns of pathogen appearance, which led them to the conclusion that the detection of major mastitis pathogens underlies spatio-temporal changes (Acharya et al. 2021).

Of the three in the present investigation most often found bacteria, NASM are generally considered minor pathogens, but they became increasingly important due to their high prevalence in bovine milk (Schukken et al. 2009). They do usually not cause clinical mastitis or changes in milk yield, but are strain-specifically and increasingly responsible for subclinical mastitis with enhanced somatic cell counts and persistent intramammary infections (Nyman et al. 2018). Therefore, it rather comes as a surprise that in the present investigation, NASM were on a downward trend since 2005. However, a major change in the applied examination methods or classification criteria can be ruled out as the underlying cause. Whilst we can only speculate about the sampling consistency, we must point out that the total number of submitted samples has also decreased over the years. A random species differentiation by MALDI-TOF-MS revealed primarily *S. chromogenes*, followed by *M. sciuri*, *S. xylosus* and *S. haemolyticus*. This is largely consistent with data from the literature, in which *S. chromogenes*, *S. simulans*, *S. haemolyticus*, *S. vitulinus*, *M. sciuri*, *S. xylosus* and *S. epidermis* are described as the most common NASM species involved in bovine intramammary infections (Ruiz-Romero and Vargas-Bello-Pérez 2023; Vanderhaeghen et al. 2014). It should be noted in this context that NASM isolates have not been routinely characterised at the species level. The biochemical differentiation between coagulase-positive and -negative isolates also has its limitations, as some NASM species such as *S. intermedius* or *S. pseudintermedius* are in fact coagulase-positive (Decristophoris et al. 2011), and there are also a few *S. aureus* strains that are coagulase-negative (Locatelli et al. 2023).

Different from NASM, the presence of *S. uberis* increased. *S. uberis* is the cause of a wide range of subclinical and clinical mastitis incidents in lactating and non-lactating cows and heifers. Increasing prevalence of *S. uberis* mastitis has also been previously described (Kerro Dego et al. 2018). Although the bacterium is considered generally highly susceptible to antimicrobials, treatments are often unsuccessful and the disease recurs. This is primarily because *S. uberis* occurs in the immediate environment of cows, especially in faeces and manure. Thus, the risk of re-infection is high, even after successful antibiotic treatment. Comprehensive hygiene measures are therefore essential in addition to antibiotic therapy of sufficient duration. *S. uberis* can also cause chronic infections and become deeply embedded in the udder tissue, making the pathogen inaccessible to antibiotic treatment (Patel et al. 2009).

The number of detected *E. coli* in our samples also increased over the last 19 years. At present, coliforms, especially *E. coli*, are a common cause of clinical mastitis in dairy cows (Hogan and Larry Smith 2003). Infection results in inflammation of the mammary gland with major local and occasionally systemic clinical symptoms (Hogan and Larry Smith 2003; Burvenich et al. 2003).

Beyond the expected mastitis pathogens, the percentage of samples contaminated with more than three different microorganisms generally remained constant throughout the last 19 years, on average at 5% of all positive samples. We also could observe differences between individual submitters (data not shown). This is also worth mentioning, as it points to the importance of improving sterile milk sampling by veterinarians and dairy farmers. With each of these results, the diagnostics laboratory of the Department of Milk Hygiene sends out a short instruction for sterile sampling. Special training courses would also be beneficial.

In summary, we showed that the most common mastitis pathogens such as NASM, *S. uberis*, *E. coli* or *S. aureus* were also present in abundance in our samples mainly obtained from the greater Hanover region, and that increasing and decreasing trends have been observed over the years. Special attention must be paid not only to alterations of the pathogen spectrum, but also in particular to changes in antimicrobial resistances. Several previous studies provide evidence of increased resistances of mastitis pathogens such as *E. coli*, *Enterobacter* spp., *Enterococcus* spp., NASM, *Pasteurella* spp., *S. agalactiae*, *S. aureus* or *S. uberis* against certain antimicrobials (Bechtold et al. 2024; Boireau et al. 2018; Bolte et al. 2020; Makovec and Ruegg 2003). Resistance trends for our data set are currently being analyzed.

## Supplementary Information

Below is the link to the electronic supplementary material.

Supplementary Material 1

## Data Availability

Data is provided within the manuscript or supplementary information files.
