# Supplementary material for: Occurrence of mastitis pathogens in cow’s milk samples from the Hanover region, north-western Germany – an overview of routine laboratory data from 2005 to 2023
Source: Vet Res Commun. 2025 May 22;49(4):208. doi: 10.1007/s11259-025-10773-1 (PMC12098181; doi:10.1007/s11259-025-10773-1)
Supplement: Supplementary file 1 — Supplementary Material 1 [file 11259_2025_10773_MOESM1_ESM.docx]

**Supplementary material**

**Occurrence of mastitis pathogens in cow's milk samples from the Hanover region, north-western Germany – An overview of routine laboratory data from 2005-2023**

Theresa Büthe^a^, Nadja Jessberger^a^*, Bettina Schneider^b^, Lothar Kreienbrock^b^ and Madeleine Plötz^a^

^a^Institute for Food Quality and Food Safety, University of Veterinary Medicine Hannover, Bischofsholer Damm 15, 30173 Hanover, Germany.

^b^Institute of Biometry, Epidemiology and Information Processing, University of Veterinary Medicine Hannover, Bünteweg 2, 30559 Hanover, Germany.

*Corresponding author: N. Jessberger, Institute for Food Quality and Food Safety, University of Veterinary Medicine Hannover, Bischofsholer Damm 15, 30173 Hanover, Germany. Phone: 0049 511 856 7547. E-mail address: Nadja.Jessberger@tiho-hannover.de.

**Supplementary Fig 1** Overview of the number and origin of samples processed at the Department of Milk Hygiene, Institute for Food Quality and Food Safety, University of Veterinary Medicine Hannover, from 2005-2023. Only cow's milk samples were included in the statistics, a total of 102,179 samples. **a** Comparison of the different groups of clients per year. **b** Geographical allocation of the samples in %. For approx. 22 % of all tested samples, no details were given on the owner. These cases were either allocated to the University of Veterinary Medicine Hannover (stationary patients at the Clinic for Cattle, or treated by the Ambulatory Veterinary Service) or to the treating veterinarian. Map of Germany: https://commons.wikimedia.org/wiki/File:Karte_gruenes_deutschland.svg. Licensing not required

**Supplementary Table 1** Overall summary for the proportion of positive findings for the 13 most common pathogen groups from 2005-2023.

| **Pathogen/Finding** | **Sample size** | **Proportion of positive findings** | | | | | | | | **Linear regression** | |
| --- | --- | --- | --- | --- | --- | --- | --- | --- | --- | --- | --- |
|  |  | **Mean** | **Median** | **STD** | **CV** | **Min** | **5%-quantile** | **95%-quantile** | **Max** | **slope** | **Pr > \|t\|** |
| **NASM** | 11,538 | 11.00 | 11.53 | 2.73 | 24.80 | 6.36 | 6.36 | 14.60 | 14.60 | -0.3443 | 0.00066 |
| ***Streptococcus uberis*** | 7,201 | 7.43 | 7.31 | 1.35 | 18.12 | 5.64 | 5.64 | 10.17 | 10.17 | 0.1272 | 0.01904 |
| ***Escherichia coli*** | 6,449 | 6.69 | 6.36 | 1.46 | 21.84 | 3.83 | 3.83 | 9.91 | 9.91 | 0.1265 | 0.03448 |
| ***Corynebacterium* spp.** | 4,650 | 3.75 | 3.47 | 2.72 | 72.50 | 0.14 | 0.14 | 7.75 | 7.75 | -0.4430 | <0.0001 |
| ***Staphylococcus aureus*** | 3,252 | 2.81 | 2.60 | 1.24 | 44.23 | 1.44 | 1.44 | 5.57 | 5.57 | -0.1988 | <0.0001 |
| **Yeast** | 2,975 | 2.80 | 2.76 | 0.63 | 22.53 | 1.44 | 1.44 | 3.79 | 3.79 | -0.0726 | 0.0027 |
| ***Enterococcus* spp.** | 2,750 | 2.36 | 2.12 | 1.20 | 50.73 | 0.82 | 0.82 | 5.27 | 5.27 | -0.1660 | <0.0001 |
| **Contaminated** | 2,434 | 2.48 | 2.35 | 1.21 | 48.85 | 0.93 | 0.93 | 6.36 | 6.36 | -0.0012 | 0.98229 |
| ***Bacillus* spp.** | 1,651 | 1.42 | 1.45 | 0.74 | 52.56 | 0.18 | 0.18 | 3.35 | 3.35 | -0.1037 | <0.0001 |
| ***Streptococcus dysgalactiae*** | 1,284 | 1.16 | 1.12 | 0.40 | 34.35 | 0.56 | 0.56 | 1.89 | 1.89 | -0.0615 | <0.0001 |
| ***Klebsiella pneumoniae*** | 738 | 0.71 | 0.71 | 0.27 | 37.68 | 0.26 | 0.26 | 1.14 | 1.14 | -0.0088 | 0.44929 |
| **Moulds** | 478 | 0.50 | 0.43 | 0.23 | 46.24 | 0.26 | 0.26 | 1.21 | 1.21 | 0.0075 | 0.45933 |
| ***Klebsiella ozaenae*** | 469 | 0.52 | 0.49 | 0.33 | 62.39 | 0.03 | 0.03 | 1.09 | 1.09 | -0.0486 | 0.01066 |
